# EANM guidelines for radionuclide therapy of bone metastases with beta-emitting radionuclides

**DOI:** 10.1007/s00259-018-3947-x

**Published:** 2018-02-16

**Authors:** Daria Handkiewicz-Junak, Thorsten D. Poeppel, Lisa Bodei, Cumali Aktolun, Samer Ezziddin, Francesco Giammarile, Roberto C. Delgado-Bolton, Michael Gabriel

**Affiliations:** 1 Nuclear Medicine and Endocrine Oncology Department, Maria Sklodowska Curie Memorial Cancer Center and Institute of Oncology, Gliwice, Poland; 2 Clinic for Nuclear Medicine, University Hospital Essen, Essen, Germany; 3 Department of Radiology, Memorial Sloan Kettering Cancer Center, New York, USA; 4 Tirocenter Nuclear Medicine Center, Istanbul, Turkey; 5 Nuclear Medicine, University Hospital USK, Saarland University, Hamburg, Germany; 6 Nuclear Medicine and Diagnostic Imaging Section, International Atomic Energy Agency (IAEA), Vienna, Austria; 7 Department of Diagnostic Imaging (Radiology) and Nuclear Medicine, University Hospital San Pedro and Centre for Biomedical Research of La Rioja (CIBIR), Logroño, La Rioja Spain; 8 Institute of Nuclear Medicine and Endocrinology, Kepler University Hospital, Linz, Austria; 9 University Clinic of Nuclear Medicine, Medical University of Innsbruck, Innsbruck, Austria

**Keywords:** Radionuclide therapy, Bone metastases, Beta-emitting radionuclides, Strontium-89, Samarium-153, Phosphorus-32, Efficacy

## Abstract

The skeleton is the most common metastatic site in patients with advanced cancer. Pain is a major healthcare problem in patients with bone metastases. Bone-seeking radionuclides that selectively accumulate in the bone are used to treat cancer-induced bone pain and to prolong survival in selected groups of cancer patients. The goals of these guidelines are to assist nuclear medicine practitioners in: (a) evaluating patients who might be candidates for radionuclide treatment of bone metastases using beta-emitting radionuclides such as strontium-89 (^89^Sr), samarium-153 (^153^Sm) lexidronam (^153^Sm-EDTMP), and phosphorus-32 (^32^P) sodium phosphate; (b) performing the treatments; and ©) understanding and evaluating the treatment outcome and side effects.

**Electronic supplementary material:**

The online version of this article (10.1007/s00259-018-3947-x) contains supplementary material, which is available to authorized users.

## Preamble

The European Association of Nuclear Medicine (EANM) is a professional nonprofit medical association that facilitates communication worldwide between individuals pursuing clinical and research excellence in nuclear medicine. The EANM was founded in 1985. EANM members are physicians, technologists, and scientists specializing in the research and practice of nuclear medicine.

The EANM periodically releases new guidelines for nuclear medicine practice to help advance the science of nuclear medicine and to improve the quality of service to patients throughout Europe. Existing practice guidelines are reviewed for revision or renewal as appropriate on their fifth anniversary or sooner if indicated. The practice guidelines on each topic, that represent policy statements by the EANM, have undergone a thorough consensus process during which they have been extensively reviewed. The EANM recognizes that the safe and effective use of diagnostic nuclear medicine imaging requires specific training, skills, and techniques, as described in each document. The EANM has written and approved these guidelines to promote the use of nuclear medicine procedures of high quality.

 These guidelines are intended to assist practitioners in providing appropriate nuclear medicine care for patients. They are not inflexible rules or requirements for practice and are not intended, nor should they be used, to establish a legal standard of care. The ultimate judgement regarding the propriety of any specific procedure or course of action must be made by medical professionals taking into account the unique circumstances of each case. Thus, there is no implication that an approach differing from the guidelines, standing alone, is below the standard of care. To the contrary, a conscientious practitioner may responsibly adopt a course of action different from that set out in the guidelines when, in the reasonable judgement of the practitioner, such a course of action is indicated by the condition of the patient, limitations of available resources, or advances in knowledge or technology subsequent to publication of the guidelines.

The practice of medicine involves not only the science but also the art of dealing with the prevention, diagnosis, alleviation and treatment of disease. The variety and complexity of human conditions make it impossible to always reach the most appropriate diagnosis or to predict with certainty a particular response to treatment. Therefore, it should be recognized that adherence to these guidelines will not ensure an accurate diagnosis or a successful outcome. All that should be expected is that the practitioner will follow a reasonable course of action based on current knowledge, available resources and the needs of the patient to deliver effective and safe medical care. The sole purpose of these guidelines is to assist practitioners in achieving this objective.

## Introduction

Metastatic bone disease is a common and severe complication of several types of advanced disease. Breast, prostate and lung cancers are collectively responsible for about 80% of secondary metastatic bone disease [1]. Pain is a major healthcare problem in patients with bone metastases. It has been reported that up to 90% of patients with metastatic or advanced cancer will experience significant cancer-related pain [2] and the majority of them will experience bone pain [3]. The spine, pelvis and ribs are often the earliest site of metastases, but most bone metastases (more than 80%) are found in the axial skeleton [4].

Treatment of cancer-induced bone pain normally progresses through the sequence: nonsteroidal analgesics to opioids often combined with radiotherapy, surgery, chemotherapy, hormone treatment, bisphosphonates and radionuclide therapy. Substantial advantages of bone radionuclide therapy include its ability to simultaneously treat multiple sites of disease, ease of administration, repeatability and potential integration with the other treatments. Not only has radionuclide therapy with alpha-emitting radionuclides (radium-223) been used to treat bone metastasis-related pain, but it has also recently been demonstrated to prolong patient survival [5].

## Goals

The goals of these guidelines are to assist nuclear medical practitioners in:

1. Evaluating patients who might be candidates for treatment of metastatic bone pain using beta-emitting radionuclides such as strontium-89 (^89^Sr, approved in Europe and the US), samarium-153 (^153^Sm) lexidronam (^153^Sm-EDTMP, approved in Europe and the US), and phosphorus-32 (^32^P sodium phosphate, approved in the US). The radionuclide radium-223 (^223^Ra-dichloride, Xofigo®, approved in Europe and the US) is an alpha emitter with different physical and clinical characteristics from those of beta emitters. It is indicated for radionuclide therapy in patients with metastatic castration-resistant prostate cancer (CRPC), symptomatic bone metastases and no known visceral metastatic disease, and separate guidelines for its use have been published [6].
2. Providing information for performing these treatments.
3. Understanding and evaluating treatment outcome and side effects.

## Methodology

These guidelines are a revised version of the previously published EANM procedure guidelines on the treatment of painful bone metastases [7]. To ensure high-quality, independent data search, a third party (the German Agency for Quality in Medicine, Germany) searched Medline via PubMed (http://ncbi.nlm.nih.gov.pubmed) and the Cochrane Library databases (http://onlinelibrary.wiley.com/cochtanelibrary/search/) to systematically locate and obtain the articles relevant to clinically used bone-seeking radiopharmaceuticals (including alpha and beta emitters for both mentioned guidelines) in metastatic bone disease (samarium-153, strontium-89, phosphorus-32, rhenium-188, radium-223) published between 2004 and 2015. Radium-223 is included, although the detailed analysis is presented in separate guidelines [6]. Tables with predefined PICO questions were used (Supplementary Table 1). The task force responsible for the guidelines also provided additional relevant articles and other materials. The following endpoints were evaluated: pain relief, the need for analgesics, adverse events, and survival. The task force members graded recommendations using criteria adopted from the US Preventive Service Task Force, from the Agency for Healthcare Research and Quality (Supplementary Table 2). The articles included in the analysis are summarized in Table 1 for strontium-89 [8–19] and Table 2 for samarium-153 [18, 20–29], and in Supplementary Table 1 [8–30].

**Table 1:** Summary of efficacy studies on strontium-89

Reference	Year	Study type	Number of patients	Dose	Cancer	Pain relief			Reduction in analgesics	Duration of response	Adverse effects	Flare phenomenon	Comments
						Scale used	Complete relief	Any relief					
[8]	1991	Single-centre, prospective randomized, double-blind, cross-over	32 (26 were evaluable)	4 mCi (150 MBq)	Prostate	Numerical weighting system	Only following strontium-89	75%	Yes	ND	Transient and slight decrease in leucocyte and platelet counts	ND	The first larger series assessing the efficacy of strontium-89
[9]	2000	Single-centre, phase I/II	40	4 mCi (148 MBq)	Breast	Nine-point scale	ND	92%	ND	120 ± 143 days	Transient and slight decrease in leucocyte and platelet counts	ND	The treatment may be repeated safely and with the same efficacy
[10]	2000	Single-centre, retrospective	94	4 mCi (150 MBq)	Prostate	Ten-point VAS	31%	78%	60%	ND	High-grade leucothrombopenia in 5%	23%	A second dose prolonged analgesia in three of four patients without increase in toxicity
[11]	2001	Single-centre, II	93	4 mCi (150 MBq)	Prostate	RTOG pain scoring system	18%	62%	ND	ND	no information	ND	Prostate-specific albumin may not provide a useful surrogate for treatment outcome
[12]	2001	Multicentre observational	527	4 mCi (148 MBq)	Prostate	RTOG pain scoring system	ND	81%	Yes	5.0 ± 3.5 months	Haematological toxicity (mild to moderate) in 25.5%	14.1%	Retreatments showed significantly worse responses than first treatments
[13]	2002	Single-centre, phase I/II, retrospective	41*	4 mCi (150 MBq)	Prostate and breast	ND	33%	81%	Yes	ND	no information	ND	None
[14]	2003	Single-centre, phase I/II	33	4 mCi (148 MBq)	Prostate, breast, bladder, and renal cell	Response index (12-point scale)	18%	88%	Yes	ND	Transient haematological toxicity in 48%	ND	Survival after therapy between 21 and 138 weeks (mean 58 weeks)
[15]	2003	Single-centre, phase II	70	4 mCi (148 MBq)	Prostate	Ten-point VAS	ND	88%	50%	ND	No information	ND	Motor activity, quality of life and Karnofsky performance score improved significantly
[16]	2003	Single-centre, phase III (comparator radiotherapy)	203	4 mCi (148 MBq)	Prostate	Five-point WHO	ND	78%	Yes	4.6 months	Haematological toxicity (grade 3/4) 1%		No differences in effectiveness; radiotherapy more gastrointestinal toxicity
[17]	2004	Single-centre, phase I/II, retrospective	13	4 mCi (148 MBq)	Prostate	Subjectively assessed by oncologist	14%	57%	Yes	56 days	Prolonged thrombocytopenia in all but one patient; leucopenia generally mild	ND	In chemotherapy-refractory prostate cancer prolonged monitoring of haematological parameters is required
[18]	2007	Single-centre, phase I/II	15	4 mCi (148 MBq)	Prostate and breast	Ten-point VAS	15%	73%	Yes	>12 weeks 46%	Thrombocytopenia mainly grade I	ND	Nadirs of platelet and leucocyte counts observed between weeks 2 and 5 after treatment and were reversible within 12 weeks
[19]	2014	Single-centre, retrospective	54	2 MBq/kg	Prostate, breast and other	Pain diary on a 0–10 numeric rating scale	34.6%	71.2%	Yes	ND	Grade 3/4: leucopenia in one patient (1.8%), neutropenia in one (1.8%), anaemia in six (11.1%), thrombocytopenia in four (7.4%)	24%	

*ND* not described, *VAS* Visual Analogue Scale, *VRS* Verbal Rating Scale, *NRS* Numeric Rating scale

*only 27 patients evaluated for efficacy

**Table 2:** Summary of efficacy studies on samarium-153

Reference	Year	Study type	Number of patients	Dose	Cancer	Pain relief			Reduction in analgesics	Duration of response	Adverse effects	Flare phenomenon	Comments
						Scale used	Complete relief	Any relief					
[20]	1993	Single-centre, phase I/II	52	0.5–3 mCi/kg (18.5–111 MBq/kg)	Prostate	Ten-point VAS	ND	67%	Yes	Mean 2.6 months	Toxicity exclusively haematological at the highest dose level; 86% recovery	ND	Patients receiving greater doses had significantly greater reductions in prostate-specific albumin
[21]	1998	Single-centre, phase II/III	118	0.5–1 mCi/kg (18.5–37 MBq/kg)	Prostate, breast, others	Area under the pain curve VAS, blinded physician’s global assessment	30%	57–65%	Yes	Through week 16 in 43% of patients	Bone marrow suppression mild, reversible and not associated with grade IV toxicity	ND	
[22]	1999	Multicentre, phase II/III	105	1 mCi/kg (37 MBq/kg)	Prostate, breast, others	Six-point scale (change in pain intensity)	About 25%	No information	87.5%	ND	Bone marrow suppression mild, reversible and not associated with grade IV toxicity	ND	Breast cancer patients showed a significant increase in Karnofsky performance score
[23]	2000	Single-centre, phase I/II	33	1 mCi/kg (37 MBq/kg)	Prostate, breast, others	ND	ND	71%	Yes	ND	no information	ND	
[24]	2003	Single-centre, phase I/II	9	1 mCi/kg (37 MBq/kg)	Prostate, breast, others	RTOG pain scoring system	0%	77.8%	ND	Pain relief maintained more than 3 weeks	Bone marrow suppression mild, reversible and not associated with grade IV toxicity	ND	
[25]	2004	Single-centre, phase II, retrospective	73	1 mCi/kg (37 MBq/kg)	Prostate, breast	0–10 point scale	ND	90% (decrease in pain score by more than 25%)	ND	ND	Mild to moderate myelosuppression noted in 75.3% of patients, recovery at 8 weeks	ND	
[26]	2004	Single-centre, phase II, retrospective	58	1.0–1.6 mCi/kg (37–59.2 MBq/kg)	Prostate, breast, others	0–10 point scale	ND	78% (decrease in pain score by more than 25%)	ND	ND	No significant myelotoxicity occurred	ND	
[27]	2004	Multicentre, phase III, prospective, randomized, double-blind (comparator - placebo)	152	1 mCi/kg (37 MBq/kg)	Prostate	0–100 VAS	38%	65%	Yes	ND	Mild, transient bone marrow suppression was the only adverse event, nadir 3 to 4 weeks after therapy, recovery after 8 weeks	ND	Complete and any pain relief significantly more frequent in radionuclide treatment group
[28]	2006	Single-centre, phase II	86	1 mCi/kg (37 MBq/kg)	Prostate, breast, others	Ten-point VAS	12%	73%	Yes	3.16 ± 1.88 months	Mild, transient bone marrow suppression was the only adverse event after therapy, recovery after 6 to 8 weeks	ND	
[29]	2007	Pilot study/case series	13	1 mCi/kg (40 MBq/kg)	Prostate	Six-point visual rating scale	31%	77%	Yes	More than 4 weeks	Mild and readily reversible in three patients	ND	
[18]	2007	Single-centre, phase I/II	15	1 mCi/kg (37 MBq/kg)	Prostate and breast	Ten-point VAS	15%	73%	Yes	>12 weeks 54%	Thrombocytopenia mainly grade I	ND	Nadirs of platelet and leucocyte counts observed between weeks 2 and 5 after treatment and were reversible within 12 weeks

*ND* not described

## Definitions

Bone metastases/Metastatic bone disease: A type of cancer metastases that results from the primary tumour disseminating and invading the bones.
Metastatic bone pain: Bone pain related to cancerous metastases located in the skeleton.
Palliative care: According to the WHO definition, this is an approach that improves the quality of life of patients and their families facing the problems associated with life-threatening illness (e.g. cancer).
Radionuclide therapy: In the context of these guidelines, this means the intravenous administration of bone-seeking radiopharmaceuticals labelled with a beta-emitting radionuclide such as strontium-89, samarium-153 or phosphorus-32 (the alpha-emitting radium-223 is addressed in detail in separate guidelines [6]).
Bone-seeking radiopharmaceuticals: Radiopharmaceuticals whose efficacy relies on selective uptake and prolonged retention at sites of increased osteoblastic activity. The exact mechanism of action is not fully understood, but may involve a reduction in pain mediators (e.g. histamine, prostaglandin E, interleukin, leukotrienes or substance P) produced by the tumour and the inflammatory cells at the interface between the tumour and normal bone and radiation-induced mechanical factors, such as a reduction in periosteal swelling [31].
Osteoblastic metastases: Focally increased skeletal metabolic activity, also termed sclerosis or sclerotic lesions, caused by an osseous reaction to bone metastases, as evidenced by increased uptake on bone scans.
Osteolytic bone lesions: Focal areas of bone destruction caused by the action of osteoclasts. A mixed pattern, however, is common in many lesions [32].

Bone-seeking radiopharmaceuticals may also be used for the treatment of primary and metastatic bone tumours, such as osteosarcoma, based on their ability to induce an osteoblastic reaction. However, this indication is not yet approved.

There are three beta-emitting radionuclides used in the treatment of painful bone metastases:

1. Strontium-89: Emits a beta minus particle with a maximum energy of 1.495 MeV, a mean energy of 0.58 MeV, an average soft tissue range of 2.4 mm and 0.00956% abundant gamma emission with a 0.91-MeV photo peak. The physical half-life is 50.5 days [33].
2. Samarium-153: Emits a beta minus particle with a maximum energy of 0.81 MeV, a mean energy of 0.23 MeV, an average soft tissue range of 0.6 mm and a 30% abundant gamma emission with a 0.103-MeV photo peak. The physical half-life is 1.94 days [34].
3. Phosphorus-32: Emits a beta minus particle with maximum energy of 1.71 MeV, a mean energy 0.70 MeV, an average soft-tissue range of 3.0 mm, and no gamma emission. The physical half-life is 14.3 days [35].

## Common clinical indications

Indications for radionuclide bone therapy with beta-emitting radionuclides include, but are not limited to, the following:

1. Painful metastatic bone lesions with osteoblastic response, as confirmed by areas of intense uptake on radionuclide bone scans.
2. Primary painful bone tumours with an osteoblastic response, as confirmed by areas of intense uptake on radionuclide bone scans. However, this indication is not yet approved.

The alpha-emitter radium-223 is indicated for the radionuclide treatment of CRPC in patients with symptomatic bone metastases and no known visceral metastatic disease. Radium-223 is the first targeted alpha therapy for this indication and provides a new treatment option. There is evidence of a significant benefit in terms of both overall survival and the time to the first symptomatic skeleton-related event. This indication is addressed in detail in separate guidelines for radium-223 [6].

## Contraindications

### Absolute

Pregnancy and breastfeeding are absolute contraindications.

### Compromised bone marrow function

In general, there is an increased risk of haematological adverse reactions such as neutropenia and thrombocytopenia in patients with evidence of compromised bone marrow reserve, e.g. following prior cytotoxic chemotherapy and/or radiation treatment (such as external beam radiation therapy, EBRT) or in patients with advanced diffuse metastatic infiltration of the bone. These patients should be treated only after careful clinical risk–benefit assessment. Close monitoring is necessary. Usually a superscan appearance on the bone scan corresponds to a major site of bone marrow involvement, and is a contraindication because of possible side effects. Moreover, therapy with bone-seeking radiopharmaceuticals cannot be recommended in this situation, since valid data on overall efficacy are not available.

A relatively low blood cell count, within certain limits, may be a relative contraindication to radionuclide bone treatment because of possible myelotoxicity. Nevertheless, the precise lower limit is not well defined in the literature and the use of granulocyte colony-stimulating factors may further lower the limit. Routinely, the following values can be considered [20, 34, 36, 37].

- **Recommendation 1.** Recommendation grade C: The following cell count limits should be applied to radionuclide treatment (except for radium-223):

1. Haemoglobin <90 g/L
2. Total white cell count <3.5 × 10^9^/L
3. Platelet count <100 × 10^9^/L

Since disseminated intravascular coagulation (DIC) may be a risk factor for severe thrombocytopenia after treatment, pretreatment clotting studies to identify patients with subclinical DIC should be performed [38].

- **Recommendation 2.** Recommendation grade A: The presence of bone marrow involvement does not represent a contraindication per se, provided that blood values remain within the cited limits and the extent of substitution does not exceed a threshold above which severe myelotoxicity is expected.
- **Recommendation 3.** Recommendation grade C: Blood cell counts should be stable before undertaking bone palliation therapy. If there is any doubt or delay in performing the therapy due to low blood cell counts, it might be worthwhile repeating blood sampling just before the treatment to exclude rapid deterioration in blood cell counts before administration of the therapeutic radionuclides.
- **Recommendation 4.** Recommendation grade C: Poor renal function reduces the plasma clearance of bone-seeking radiopharmaceuticals, resulting in a higher whole-body dose and greater risk of myelotoxicity. Therefore, patients with severely reduced renal function, i.e. creatinine >180 μmol/L and/or glomerular filtration rate <30 mL/min, should be excluded from radionuclide bone treatment.

### Life expectancy

Considering the latency in the onset of the palliative effect (from a few days to 4 weeks), radionuclide therapy is more beneficial in patients with a relatively long life expectancy and in earlier stages of metastatic bone disease [37].

- **Recommendation 5.** Recommendation grade C: Palliative therapy with strontium-89, samarium-153 or phosphorus-32 is inappropriate in patients with a life expectancy of less than 4 weeks. Life expectancy should preferably be greater than 3 months.

## Efficacy of radionuclide treatment

### Pain control

There is clinical evidence to support a beneficial effect of radionuclide therapy in patients with osteoblastic or mixed pattern (osteoblastic/osteoclastic) metastases. Review of the data published in clinical trials suggests that any pain relief can be achieved in about 50–90% of patients, including complete relief in about 12–33% (Tables 1 and 2, and Supplementary Table 3).

A systematic review and meta-analysis included 57 studies: 9 randomized clinical trials, 13 clinical trials, and 35 observational studies [39]. Most of the studies evaluated prostate cancer patients with bone metastases. The meta-analysis provided evidence that pain relief is achieved after a single radionuclide therapy in about 70% of patients (95% CI 65–75%, *p* < 0.000), 70% (95% CI 63–77%, *p* < 0.000) for strontium-89 and 70% (95% CI 63–96%, *p* < 0.000) for samarium-153. Combination with other therapies is slightly more effective: pain relief was achieved in 74% (95% CI 59–88%, *p* < 0.000). Pain relief in patients with prostate cancer was 70% (95% CI 62–76%, *p* < 0.000) and in patients with breast cancer was 79% (95% CI 72–84%, *p* < 0.000).

These results were basically confirmed by two other meta-analyses [40, 41]. A comprehensive analysis by Finlay et al. of the efficacy of different radiopharmaceuticals including prospective studies with strontium-89 (16 studies), samarium-153 (number not mentioned) and rhenium-188 (4 studies), showed complete symptomatic responses in 32% of patients (range 8–77%) and partial responses in 44% of patients. No pain palliation was used in 25% (range 14–52%). Analgesic use (poorly reported) was reduced in 71–81%. Analgesic effects were initially observed 4–28 days after therapy and the duration of response was up to 15 months [37].

A systematic review of pain relief in patients with metastatic breast cancer [42] included three randomized clinical trials, of which two compared two different radionuclides, and one compared two different levels of samarium-153 activity [43–45]. In addition, there were 16 uncontrolled trials (see 

Christensen and Petersen [42] for references). According to the Centre of Evidence-based Medicine criteria, there is level 4 evidence for the efficacy of radionuclides in bone metastasis pain palliation in patients with breast cancer. Although the majority of studies showed positive bone pain palliation effects and improvements in performance status, the conclusion was critical in terms of supporting the clinical effect of radionuclides in relieving pain from bone metastasis in patients with breast cancer.

- **Recommendation 6.** Recommendation grade A: Radionuclide therapy can be recommended as a palliative treatment in patients with painful bone metastases with osteoblastic or mixed pattern (osteoblastic/osteoclastic) features.

### Quality of life

A few studies have shown improved quality of life after radionuclide treatment for painful bone metastases [15, 46, 47].

- **Recommendation 7.** Recommendation grade B: Radionuclide treatment can be recommended to improve the quality of life in patients with osteoblastic or mixed pattern (osteoblastic/osteoclastic) bone metastases.

### Survival

There are no studies that have investigated survival benefits after radionuclide therapy with beta-emitting radionuclides such as strontium-89, samarium-153 or phosphorus-32. However, a phase II study in prostate cancer showed a survival benefit if chemotherapy (doxorubicin) was added to strontium-89 (27.7 vs. 16.8 months) [48]. On the other hand, recent studies with radium-223 have shown improved overall survival in patients with metastatic CRPC and bone metastases without visceral dissemination [5, 49]. Although this topic is addressed in detail in the separate guidelines on radium-223 [6], given that there is increased survival benefit we have also included this recommendation here.

- **Recommendation 8.** Recommendation grade A: Radionuclide treatment can be recommended to prolong survival only with the alpha-emitting radium-223 in prostate cancer patients with osteoblastic or mixed pattern (osteoblastic/osteoclastic) bone metastases without visceral dissemination (for reference see the EANM guidelines for radionuclide therapy with radium-223 of metastatic CRPC [6]). There is no evidence that other therapeutic radionuclides improve overall survival.

## Efficacy of other bone metastasis treatments and their combination with radionuclide therapy

### External beam radiotherapy

A systematic overview of radiation therapy performed by the Swedish Council of Technology Assessment in Health Care (SBU in its Swedish abbreviation) provided strong evidence that radiotherapy of skeletal metastases provides an overall response (complete and partial pain relief) in more than 80% of patients. Furthermore, this study showed that the duration of pain relief in at least 50% of patients persists for ≥6 months. External hemi-body radiation performed in patients with numerous painful bone metastases can result in severe bone marrow suppression. Therefore, to reduce the probability of synergistic myelotoxic effects between external hemi-body radiation and radionuclide administration, each patient should be carefully evaluated.

EBRT is the treatment of choice if the bone scan is negative. In patients with impending pathological fracture, tele-radiotherapy (and/or surgical intervention) is required [50]. Since the evidence in published reports is contradictory [46, 51], a combination of EBRT and radionuclide therapy should be used only in selected patients (e.g. those with predominant and severe pain in one of multiple painful metastatic foci).

- **Recommendation 9**. Recommendation grade C: Concomitant or sequential radionuclide and EBRT can be used In selected patients for the treatment of painful osteoblastic or mixed pattern (osteoblastic/osteoclastic) bone metastases.

### Bisphosphonate treatment

Bisphosphonates decrease bone resorption and increase mineralization by specifically inhibiting osteoclastic activity. Multiple, randomized, controlled trials have clearly demonstrated that they are effective in reducing skeletal morbidity and pain from metastatic cancer [52, 53]. There are conflicting data as to whether bisphosphonates inhibit the uptake of radiolabelled phosphonates in bone metastases. Recent studies have shown no evidence of competition between bisphosphonates and samarium-153 or strontium-89 [54–57]. Therefore, they may be used concomitantly or sequentially.

- **Recommendation 10.** Recommendation grade B: There are no contraindications for concomitant or sequential use of radionuclide therapy and bisphosphonates for the treatment of patients with painful osteoblastic or mixed pattern (osteoblastic/osteoclastic) bone metastases.

Interactions with calcium, phosphate and vitamin D cannot be excluded due to physiological relationships.

### Chemotherapy

Several studies have shown the effectiveness of chemotherapy in patients with hormone-refractory prostate cancer in terms of pain palliation. For example, chemotherapy with docetaxel every 3 weeks plus prednisone leads to better survival and also improved response rates in terms of pain, serum prostate-specific albumin levels, and quality of life [58]. In a phase III trial, Basch et al. found that abiraterone acetate plus prednisone delays patient-reported pain progression in chemotherapy-naive patients with metastatic CRPC [59].

There are no data supporting the view that concomitant or sequential use of radionuclide and chemotherapy increases palliative efficacy. However, because of potentially severe leucopenia or thrombocytopenia, patients should not have received long-acting myelosuppressive chemotherapy (e.g. nitrosoureas) for 6–8 weeks and other forms of myelosuppressive treatment or systemic radioisotope therapy for approximately 4 weeks prior to the administration of strontium-89, samarium-153 or phosphorus-32. After strontium-89, samarium-153 or phosphorus-32 administration, therapeutic administration of myelosuppressive systemic treatments should be withheld for about 12 weeks. Nonmyelosuppressive medical therapies (including hormone therapy in breast/prostate cancer) should not be interrupted before strontium-89, samarium-153 or phosphorus-32 administration.

Due to the limited data available with respect to the combination of bone-seeking radiopharmaceuticals and kinase inhibitors, this application is only recommended with reservations.

- **Recommendation 11.** Recommendation grade D: Concomitant radionuclide therapy and chemotherapy should be used carefully because of possible haematological side effects. Current evidence on the bone palliation efficacy of the concomitant use of radionuclide therapy and chemotherapy is inconclusive.

## Qualifications and responsibilities of personnel

Radionuclide bone treatment in patients with metastatic bone disease must be performed by a multidisciplinary team that should include a nuclear medicine physician, a medical oncologist, a radiation oncologist, and, as necessary, a medical physicist experienced in nuclear medicine procedures. The mandatory procedures to be undertaken prior to strontium-89, samarium-153 or phosphorus-32 administration are summarized in Table 3.

**Table 3:** Mandatory procedures to be performed before strontium-89, samarium-153, phosphorus-32 or radium-223 administration

Procedure	Objective	Timing
Medical history	To obtain patient demographics, indication for therapy, concomitant medications	Qualification for treatment on day of treatment
Life expectancy estimation	To confirm at least 4–6 weeks (preferably 3 months)	Qualification for treatment
Bone scan	To evaluate extent of disease	No longer than 4–8 weeks prior to therapy
Radiological imaging	To exclude severe lytic lesions with risk of pathological bone fracture or cord compression	As required
Complete blood count, d-dimer, serum creatinine	To exclude haematological, biochemical contraindication to therapy	No longer than 1–2 weeks prior to therapy; If required repeat on day of treatment
Pregnancy test		On day of treatment

Bone-seeking radiopharmaceuticals labelled with beta-emitting radionuclides (strontium-89, samarium-153, phosphorus-32) should be administered only by an appropriately trained and certified nuclear medicine physician in a facility licensed to use these radioactive materials. The licence should be compatible with national legislation. If in-patient treatment is required by national legislation, this should take place in an approved facility with appropriately shielded rooms and en-suite bathroom facilities. The facility in which treatment is administered must have appropriately trained personnel, radiation safety equipment, and clearly defined procedures for waste handling and disposal, handling of contamination, monitoring of personnel for accidental contamination and controlling contamination spread [36].

## Examination procedure/specifications

### Request

The patient medical history should be obtained with special emphasis on severity, localization and duration of bone pain and its response to other treatment modalities. Prior to the administration of strontium-89, samarium-153 or phosphorus-32, the patient should have had a recent bone scan (within the previous 8 weeks) documenting increased osteoblastic activity at the painful sites. Radiographs demonstrating osteosclerotic lesions are not adequate, as increased bone density does not always result in increased uptake on radionuclide imaging. Abnormalities on the bone scan must be correlated with an appropriate physical examination to exclude other causes of chronic pain, which would be unlikely to respond to treatment using bone-seeking radiopharmaceuticals. Neurogenic pain and pathological fractures should be specifically excluded. Bone scintigraphic abnormalities should also be correlated with the physical examination and other imaging studies to ascertain that osseous or soft-tissue abnormalities, which might cause cord or nerve compression or pathological fractures, are not present. The only indication for the use of strontium-89, samarium-153 or phosphorus-32 in these circumstances would be in conjunction with local treatment, either radiation therapy or surgical intervention, if there are other sites of painful bone metastases.

A full haematological and biochemical profile should be obtained during the 7 days before the proposed treatment. Recommended reference for haematological and biochemical levels are listed in recommendations 1to 4.

### Patient preparation and precautions

In the absence of contraindications for strontium-89, samarium-153 or phosphorus-32 radionuclide therapy, there is no special patient preparation required prior to treatment. Patients should receive information pertaining to the procedure prior to receiving the therapy. Written informed consent must be obtained from the patient, if required by local legislation. It should be explained to the patient that phosphorus-32, strontium-89 or samarium-153 are radionuclide treatments specifically designed for treating bone pain. Patients should be informed that 50–90% of patients benefit from phosphorus-32, strontium-89 or samarium-153 therapy. Patients should also be warned about the risk of a temporary increase in bone pain (pain flare) and that pain reduction is unlikely to occur within the first week after the treatment, is more likely to occur in the second week and could occur as late as 4 weeks or longer after injection, particularly for long-lived radionuclides. Patients should continue to take prescribed analgesics until bone pain decreases and receive advice regarding subsequent analgesic dose reduction where appropriate. Patients should also be informed about the duration of the analgesic effect, which is generally of 2–6 months, and that retreatment is possible.

### Radiopharmaceutical administration

Strontium-89, samarium-153 and phosphorus-32 are supplied in a solution to be used at room temperature. They should be administered by slow infusion via an indwelling intravenous butterfly or cannula followed by a 0.9% saline flush. Care should be taken to avoid extravasation of the radiopharmaceutical, and catheter patency should always be checked before infusion. If extravasation is noticed, infusion should be stopped and as much radiopharmaceutical as possible should be withdrawn. There are very limited data regarding the procedure, but cooling the site of extravasation can prevent radionuclide spread. In case of circulatory or nerve impairment, surgery may be indicated [60].

The radiopharmaceutical should be injected by a certified nurse under the responsibility of a certified nuclear medicine physician according to national laws which may differ slightly among countries. According to EU Directive 2013/59 (art. 56.4 and art. 60.1), activities should be individually measured using a properly calibrated activity meter before administration. Recommended administered activities are as follows:

Strontium-89 (^89^Sr): 150 MBq (1.5–2.2 MBq/kg)

Samarium-153 (^153^Sm) lexidronam: 37 MBq/kg

Phosphorus-32: 185–370 MBq administered intravenously, 370–444 MBq administered orally.

Although no clear differences in treatment response among phosphorus-32, strontium-89 and samarium-153 have been reported, there are differences in the onset and duration of the responses. Patients with progressive disease and pain, in whom rapid relief is warranted, are best treated with short-lived isotopes. Relief will be quick and toxicity acceptable [60]. Patients with pain relief after radionuclide treatment, in whom pain recurs, can be re-treated unless there are contraindications for the therapy (see section Contraindications). Patients with a somewhat better prognosis and better clinical condition may be treated with long-lived radionuclides. The duration of response will be longer. However, the possibility of increased myelosuppressive toxicity must be born in mind.

## Side effects and radiation safety

### Side effects

The “flare” phenomenon involves an increase in pain symptoms. It usually occurs within 72 h of initiating treatment and is seen in about 10% of patients. In the majority of patients it is mild and self-limiting and usually responds to standard analgesics. Generally, a flare phenomenon is associated with a good clinical response [33, 37, 45, 59]. The presence of cervicodorsal spinal metastases may be associated with increased risk of spinal cord compression. Prophylactic corticosteroids may be considered, and spinal MRI and/or a neurological consultation is recommended before treatment.

Decreases in thrombocyte and leucocyte counts in the peripheral blood as a result of myelosuppression are frequently observed and have a nadir at 3–5 weeks (samarium-153) or 12–16 weeks (strontium-89 and phosphorus-32). The occurrence of grade 3 or 4 toxicity is dependent on previous (myelosuppressive) therapy and bone marrow reserve. Haematological toxicity is usually temporary, with complete or partial recovery within 3 months. Periodical haematological monitoring may be useful for up to 6 weeks after treatment (samarium-153) to exclude significant myelosuppression in high-risk patients. After treatment with strontium-89 and phosphorus-32, longer follow-up is necessary because of prolonged myelosuppressive toxicity (12–16 weeks) [35].

A flushing sensation similar to that seen with calcium infusion has been reported to occur with strontium-89 infusion, but can avoided if the compound is infused slowly, as recommended [61].

### Radiation safety

The treating physician must advise the patient on measures to reduce unnecessary radiation exposure to family members and the public. Following treatment, patients must avoid pregnancy for at least 6 months after treatment with phosphorus-32, strontium-89 or samarium-153. Patients should be appropriately hydrated before and after treatment. If the treatment is performed on an outpatient basis, patients should remain in the nuclear medicine facility for 4–6 h after administration to assess any early side effects. Urinary radiopharmaceutical excretion is of particular concern during the first 2 or 3 days. For samarium-153, it is nearly complete during the first 8–12 h after administration. Patients should be advised to observe rigorous hygiene to avoid contaminating groups at risk using the same toilet facility. Patients should be warned to avoid soiling underclothing or areas around the toilet bowl for 1 week after injection (2 or 3 days are enough for samarium-153), and that significantly soiled clothing should be washed separately. A double toilet flush is recommended after urination. Patients should wash their hands after urination. If contaminated with urine, patients should wash their hands abundantly with cold water and soap without scrubbing [37].

Incontinent patients should be catheterized before radiopharmaceutical administration for the radioprotection of relatives and/or carers. The catheter should remain in place for an appropriate period (4 days for strontium-89; 24 h for samarium-153). Catheter bags should be emptied frequently. Those caring for catheterized patients should wear gloves. If inpatient treatment is required, nursing personnel must be instructed in radiation safety. Any significant medical conditions should be noted and contingency plans must be in place to cover the eventuality that radiation precautions have to be breached because of a medical emergency. Concern about radiation exposure should not interfere with prompt appropriate medical treatment of the patient.

## Dosimetry

### Strontium-89: ^89^Sr-strontium-chloride 

Labelling: The radiopharmaceutical is supplied in aqueous solution.

The dosimetry of strontium-89 is presented in Table 4.

**Table 4:** Dosimetry of strontium-89: ^89^Sr-strontium-chloride [62]

Organ	Absorbed dose per administered activity (mGy/MBq)
Bone surface	17.0
Red bone marrow	11.0
Lower bowel wall	4.7
Bladder wall	1.3
Testes	0.8
Ovaries	0.8
Uterus wall	0.8
Kidneys	0.8

### Samarium-153: samarium (^153^Sm) lexidronam (^153^Sm-EDTMP)

Labelling: The radiopharmaceutical is supplied frozen in aqueous solution.

The dosimetry of strontium-89 [63] is presented in Table 5.

**Table 5:** Dosimetry of samarium-153: samarium (^153^Sm) lexidronam (^153^Sm-EDTMP) [63]

Organ	Absorbed dose per administered activity (mGy/MBq)
Bone surface	6.8
Red bone marrow	1.5
Lower bowel wall	0.01
Bladder wall	1.0
Testes	0.005
Ovaries	0.009
Kidneys	0.02

### Phosphorus-32

The dosimetry of phosphorus-32 is presented in Table 6.

**Table 6:** Dosimetry of phosphorus-32 [62]

Organ	Absorbed dose per administered activity (mGy/MBq)
Bone surface	11.0
Red bone marrow	11.0
Lower bowel wall	0.74
Bladder wall	0.74
Testes	0.74
Ovaries	0.74
Uterus wall	0.74
Kidneys	0.74

### Radium-223

For reference see the EANM guidelines for radionuclide therapy with radium-223 [6].

## Electronic supplementary material

ESM 1 (DOCX 27 kb)
