# Supplementary material for: EANM guidelines for radionuclide therapy of bone metastases with beta-emitting radionuclides
Source: Eur J Nucl Med Mol Imaging. 2018 Feb 16;45(5):846–59. doi: 10.1007/s00259-018-3947-x (PMC5978928; doi:10.1007/s00259-018-3947-x)
Supplement: Supplementary file 1 — (DOCX 27 kb) [file 259_2018_3947_MOESM1_ESM.docx]

**SUPPLEMENTARY MATERIAL**

**Supplementary TABLE 1**. PICO Table for the Bone therapy and Bone Palliation Guidelines (search period: 10 years up to the end December 2015)

| **No.** | **Patients** | **Intervention** | **Comparison** | **Outcome** | **Study type** |
| --- | --- | --- | --- | --- | --- |
| 1 | Patients with prostate cancer and diffuse painful bone metastasis | Radionuclide therapy (application of samarium-153, strontium-89, rhenium-186,  radium-223) | 1. Placebo  2. Non-steroidal anti-inflammatory and opioids  3. External radiation therapy (local)  4. Comparison of radionuclides be-tween each other  5. Biphosphonates | 1. Percentage of pain relief  2. Duration of pain relief (median)  3. Adverse events (myelo-supression)  4. Need for analgesics  5. Disease control  6. Survival | RCT, systematic review, meta-analysis |
| 2 | Patients with breast cancer and diffuse painful bone metastasis | Radionuclide therapy (application of samar-ium-153, strontium-89, rhenium-186,  radium-223) | 1. Placebo  2. Non-steroidal anti-inflammatory and opioids  3. External radiation therapy  (local)  4. Comparison of radionuclides  5. Biphosphonates | 1. Percentage of pain relief  2. Duration of pain relief (median)  3. Adverse events (myelo-supression)  4. Need for analgesics  5. Disease control  6. Survival | RCT, systematic review, meta-analysis |
| 3 | Patients with diffuse bone metastasis (excluding prostate and breast) | Radionuclide therapy (application of samarium-153, strontium-89, rhenium-186) |  | 1. Percentage of pain relief  2. Duration of pain relief (median)  3. Adverse events (myelo-supression)  4. Need for analgesics | Cohort studies |

Presentation in evidence Tables: For the identified publications the SIGN checklists and SIGN grading for assignment of level of evidence was used.

**Supplementary TABLE 2**. Levels of evidence grading system (adapted from United States Preventive Service Task Force)

| **Grade of recommendation** | **Methodological quality of supporting evidence** |
| --- | --- |
| **A Strong recommendation** | Randomized clinical trials (RCTs) or strong evidence from observational studies |
| **B Recommendation** | Observational studies or case series |
| **C Recommendation** | Based on expert opinion and patient preferences |
| **D Recommendation against** | Moderate or high certainty that the service has no net benefit or that the harms outweigh the benefits |
| **I Recommends neither for nor against** | Current evidence is insufficient to assess the balance of benefits and harms of the service |

**Supplementary TABLE 3.** Summary of efficacy studies on strontium-89 and samarium-153 in the past years

| **Summary of efficacy studies on strontium-89 and samarium-153 in the past years** | | | | | | | | | | | | | |
| --- | --- | --- | --- | --- | --- | --- | --- | --- | --- | --- | --- | --- | --- |
|  | **References** | **Year** | Study type | **No. of patients** | **Dose (SI)** | **Cancer** | **Pain relief %** | **Reduction of analgesics** | **Duration of response** | **Adverse effects** | **Flare phenomenon** | **Objective response criteria** | **Comments** |
| **Strontium-89** | Fuster et al. [9] | 2000 | single-centre, I–II | 40 | 4 mCi (148 MBq) | Breast | 92% | no information | 120±143 days | transient and slight decrease of leukocyte and platelet counts | no information | no information | the treatment may be repeated safely and with the same efficacy |
|  | [Kraeber-Bodere et al. [10]](file:///C:\Users\Daria\Desktop\Daria\publikacje%20różne\EANM%20SNM%20guidelines\revision_wysłane_19_10_2017\Supplementary_table_3.xls#RANGE!bib43) | 2000 | single-centre, retrospective | 94 | 4 mCi (150 MBq) | Prostate | 78% | 60% | no information | high-grade leukothrombopenias in 5% | 23% | no information | a second dose prolonged analgesia in 3/4 of cases without increase of toxicity |
|  | Turner et al. [11] | 2001 | single-centre, II | 93 | 4 mCi (150 MBq) | Prostate | 63% | no information | no information | no information | no information | no information | PSA may not provide a useful surrogate for treatment outcome |
|  | [Dafermou et al. [12]](file:///C:\Users\Daria\Desktop\Daria\publikacje%20różne\EANM%20SNM%20guidelines\revision_wysłane_19_10_2017\Supplementary_table_3.xls#RANGE!bib52) | 2001 | multi-centre III | 527 | 4 mCi (148 MBq) | Prostate | 59.80% | yes | 5.0±3.5 months | haematological toxicity (mild to moderate) in 25.5% | 14.1% | no information | retreatments showed significantly worse responses than first treatments |
|  | [Ashayeri et al. [13]](file:///C:\Users\Daria\Desktop\Daria\publikacje%20różne\EANM%20SNM%20guidelines\revision_wysłane_19_10_2017\Supplementary_table_3.xls#RANGE!bib53) | 2002 | single-centre, I–II, retrospective | 27 | 4 mCi (150 MBq) | Prostate and breast | 81% | yes | no information | no information | no information | no information | no |
|  | Zorga et al. [14] | 2003 | single-centre, I–II | 33 | 4 mCi (148 MBq) | Prostate, breast, bladder, and renal cell | 82% | yes | no information | transient haemotoxicity was observed in 48% | no information | no information | duration of life after therapy was between 21 to 138 weeks (mean 58 weeks) |
|  | Baczyk et al. [15] | 2003 | single-centre, II | 70 | 4 mCi (148 MBq) | Prostate | 88% | 50% | no information | no information | no information | no information | the motor activity, QoL and Karnofsky improved significantly |
|  | Gunawardana et al. [17] | 2004 | single-centre, I–II | 13 | 4 mCi (148 MBq) | Prostate | 57% | yes | 56 days | prolonged thrompocytopenia in all but one patient, leukopenia was generally mild | no information | no information | in chemotherapy-refractory prostate cancer prolonged monitoring of hematologic parameters is required |
|  | Liepe K et al. [18] | 2007 | single-centre, I–II | 15 | 4 mCi (148 MBq) | Prostate and breast | 72% | yes | no information | thrompocytopenia mainly grade I | no information | no information | the maximum nadir of platelet and leukocyte counts were observed between the 2nd to 5th week after treatment and was reversible within 12 weeks |
| **Samarium-153** | Collins et al. [20] | 1993 | single-centre, I–II | 52 | 0.5–3 mCi/kg (18.5–111 MBq/kg) | Prostate | 75% | yes | mean duration 2.6 months | toxicity was exclusively hematologic at the highest dose level, 86% recovery | no information | patients receiving greater doses showed a trend toward improved survival | patients receiving greater doses shad significantly greater reductions in PSA |
|  | Serafini et al. [21] | 1998 | single-centre, II–III | 118 | 0.5–1 mCi/kg (18.5-37 MBq/kg) | Prostate, breast, others | 62%–72% | yes | through week 16 in 43% of patients | bone marrow suppression was mild, reversible and not associated with grade IV tox. | no information | no information |  |
|  | Tian et al. [22] | 1999 | multi-centre II–III | 105 | 1 mCi/kg (37 MBq/kg) | Prostate, breast, others | 84% | 87.5% | no information | bone marrow suppression was mild, reversible and not associated with grade IV tox. | no information | no information | only breast cancer patients showed a significant change in Karnofsky score |
|  | Dolezal et al. [23] | 2000 | single-centre, I–II | 33 | 1 mCi/kg (37 MBq/kg) | Prostate, breast, others | 71% | yes | no information | no information | no information | no information |  |
|  | Wang et al. [24] | 2003 | single-centre, I–II | 9 | 1 mCi/kg (37 MBq/kg) | Prostate, breast, others | 78% | no information | pain relief maintained more than 3 weeks | bone marrow supression was mild, reversible and not associated with grade IV toxicity | no information | no information |  |
|  | Sapienza et al. [25] | 2004 | single-centre, II, retrospective | 73 | 1 mCi/kg (37 MBq/kg) | Prostate, breast | 76% | no information | no information | mild to moderate myelosupression was noted in 75.3% of pat, recovery at 8 weeks | no information | no information |  |
|  | Etchebehere et al. [26] | 2004 | single-centre, II, retrospective | 58 | 1.0–1.6 mCi/kg (37–59.2 MBq/kg) | Prostate, breast, others | 78% | no information | no information | no significant myelotoxicity occured | no information | no information |  |
|  | Sartor et al. [27] | 2004 | multi-centre III, prospective, randomized, double-blinded | 152 | 1 mCi/kg (37 MBq/kg) | Prostate | 65% | yes | no information | mild, transient bone marrow supression was the only adverse event, nadir 3 to 4 weeks after therapy, recovery after 8 weeks | no information | no information |  |
|  | Tripathi et al. [28] | 2006 | single-centre, II | 86 | 1 mCi/kg (37 MBq/kg) | Prostate, breast, others | 73% | yes | conducted regularely for 16 weeks | mild, transient bone marrow supression was the only adverse event after therapy, recovery after 6 to 8 weeks | no information | no information |  |
|  | Ripamonti et al. [29] | 2007 | Pilot study/ Case series | 13 | 1 mCi/kg (40 MBq/kg) | Prostate | 61.50% | yes | more than 4 weeks | mild and readily reversible in 3 patients | no information | no information |  |
|  | Liepe K et al. [18] | 2007 | single-centre, I–II | 15 | 1 mCi/kg (37 MBq/kg) | Prostate and breast | 73% | yes | no information | thrompocytopenia mainly grade I | no information | no information | the maximum nadir of platelet and leukocyte counts were observed between the 2nd to 5th week after treatment and was reversible within 12 weeks |
|  | Olea et al. [30] | 1996 | single-centre, I–II | 40 | 1.0–1.5 mCi/kg (37-55 MBq/kg) | Prostate, breast, others | 67.5% | no information | no information | bone marrow depression was observed in 37% of them | no information | no information | in 17 patients more than one dose was injected |
|  | | | | | | | | | | | | | |
